# The genetic overlap between osteoporosis and craniosynostosis

**DOI:** 10.3389/fendo.2022.1020821

**Published:** 2022-09-26

**Authors:** Erika Kague, Carolina Medina-Gomez, Simeon A. Boyadjiev, Fernando Rivadeneira

**Affiliations:** ^1^ School of Physiology, Pharmacology and Neuroscience, Biomedical Sciences, University of Bristol, Bristol, United Kingdom; ^2^ Department of Internal Medicine, Erasmus Medical Center (MC), University Medical Center Rotterdam, Rotterdam, Netherlands; ^3^ Department of Pediatrics, University of California, Davis, Sacramento, CA, United States; ^4^ Department of Oral and Maxillofacial Surgery, Erasmus Medical Center (MC), University Medical Center Rotterdam, Rotterdam, Netherlands

**Keywords:** craniosynostosis, zebrafish, fractures, bone mineral density, genome-wide association studies, osteoporosis

## Abstract

Osteoporosis is the most prevalent bone condition in the ageing population. This systemic disease is characterized by microarchitectural deterioration of bone, leading to increased fracture risk. In the past 15 years, genome-wide association studies (GWAS), have pinpointed hundreds of loci associated with bone mineral density (BMD), helping elucidate the underlying molecular mechanisms and genetic architecture of fracture risk. However, the challenge remains in pinpointing causative genes driving GWAS signals as a pivotal step to drawing the translational therapeutic roadmap. Recently, a skull BMD-GWAS uncovered an intriguing intersection with craniosynostosis, a congenital anomaly due to premature suture fusion in the skull. Here, we recapitulate the genetic contribution to both osteoporosis and craniosynostosis, describing the biological underpinnings of this overlap and using zebrafish models to leverage the functional investigation of genes associated with skull development and systemic skeletal homeostasis.

## Introduction

Osteoporosis is characterized by low bone mass, microarchitectural deterioration, and impairment of bone quality, predisposing individuals to fractures (1). Although fragility fractures caused by osteoporosis may not lead to immediate death, they have a big impact on the quality of life of patients and bring on a considerable burden to the health and economic systems. Fractures cause substantial pain, disability, loss of independence and are associated with a 20% mortality rate within one-year post-fracture (2).

Osteoporosis is the most prevalent metabolic bone disease, with estimates that 1 in 3 women and 1 in 5 men over the age of 50 will experience fragility fractures in their remaining lifetimes. Therefore, it is a major health problem as the aging population increases worldwide. In the United States, the direct medical costs of osteoporosis-related fractures alone exceed $20 billion annually (3). More than 500,000 fractures due to osteoporosis happen every year in the UK alone, representing treatment costs of over £4 billion per year (1). In Europe, osteoporosis causes 4.3 million fragility fractures per year, costing the health care systems more than 56 billion euros each year (The International Osteoporosis Foundation, SCOPE 2021). Given such alarming numbers, the World Health Organization (WHO) considers osteoporosis a leading health problem worldwide, with a burden only exceeded by cardiovascular diseases. However, despite the severe consequences of the increment of fracture number in elderly patients worldwide and the availability of diverse therapeutic options, there is still limited and in some instances, inadequate adherence of patients to treatments (described as the “gap in osteoporosis treatment”). This is a multifactorial phenomenon where limitations to long-term therapies, response to treatment and fear of adverse events contribute importantly. Altogether there is room for the identification of new biologic pathways and factors that constitute new therapeutic targets, which begins through gaining a better understanding of the underlying molecular mechanisms.

The causes of osteoporosis are attributed to a complex interplay between genetic and environmental factors. For instance, physical activity, smoking, alcohol use, and diet (calcium and protein intake) have been shown to influence the risk of osteoporotic fractures (4). Yet, twin and family studies have demonstrated that osteoporosis is highly heritable, and genetic factors are solid contributors to the risk of disease. Indeed, a wide range of osteoporosis-related phenotypes have been robustly associated with genetic factors, including markers of bone turnover, skeletal dimensions, bone growth, bone mineral density (BMD), and fracture risk itself (5–8). Among these, BMD measured using dual-energy X-ray absorptiometry (DXA), which is used to diagnose osteoporosis and assess fracture risk (9), has the highest heritability (ranging between 50% to 80%) (10). Therefore, most genetic studies in osteoporosis have focused on the BMD phenotype.

Over the years, genome wide-association studies (GWAS) have uncovered over 500 genomic loci associated with BMD. Beyond the contribution to our understanding of molecular mechanisms in bone biology these findings might frame the discovery of new therapeutic targets for osteoporosis (11, 12). In fact, the success rate during a drug’s development is doubled for drug targets with human genetic support (13). Nevertheless, GWASs have not yet contributed to translational therapeutic approaches for bone conditions. This is highly attributed to mapping the association signals and identifying the causal genes. Bone diseases with a known genetic cause help interpret a gene’s function, as learned from many cases of monogenic disorders (14). However, functional evidence from animal models provides crucial causality evidence in most cases. Great initiatives in mouse models, such as the “Origins of Bone and Cartilage Disease” (OBCD) and the “International Mouse Phenotyping Consortium” (IMPC), have helped to functional annotate GWAS candidate genes (12, 15). But, given the large number of BMD-associated loci, mouse models still fall behind in providing high-throughput evidence of implication for the numerous genes within an associated locus harmonically, rapidly, and longitudinally.

BMD is a widely available measurement of bone strength, resulting in a large yield of associated loci that have helped characterize the genetic architecture of osteoporosis during the last decade. In addition, is an excellent trait suitable for the identification of a wide spectrum of bone biology, relevant to many other bone conditions, such as osteogenesis imperfecta, sclerosteosis, and Paget’s disease of bone (16, 17), with diverse, overlapping loci. Recently, an exciting overlap in associated signals between BMD and craniosynostosis has become evident. Craniosynostosis is a developmental condition that affects the skull shape and volume as a consequence of premature fusion of the calvarial sutures (18). In this review, we will briefly discuss the genetics of osteoporosis, its overlap with craniosynostosis, and will explore the implications of this intriguing intersection. Furthermore, we will introduce zebrafish as a model organism that allows versatile functional studies aimed at the identification of causal genes from GWAS, and specifically to understand the observed overlap between these two diseases.

## A brief walkthrough of 15 years of GWAS of osteoporosis

**Figure 1:**
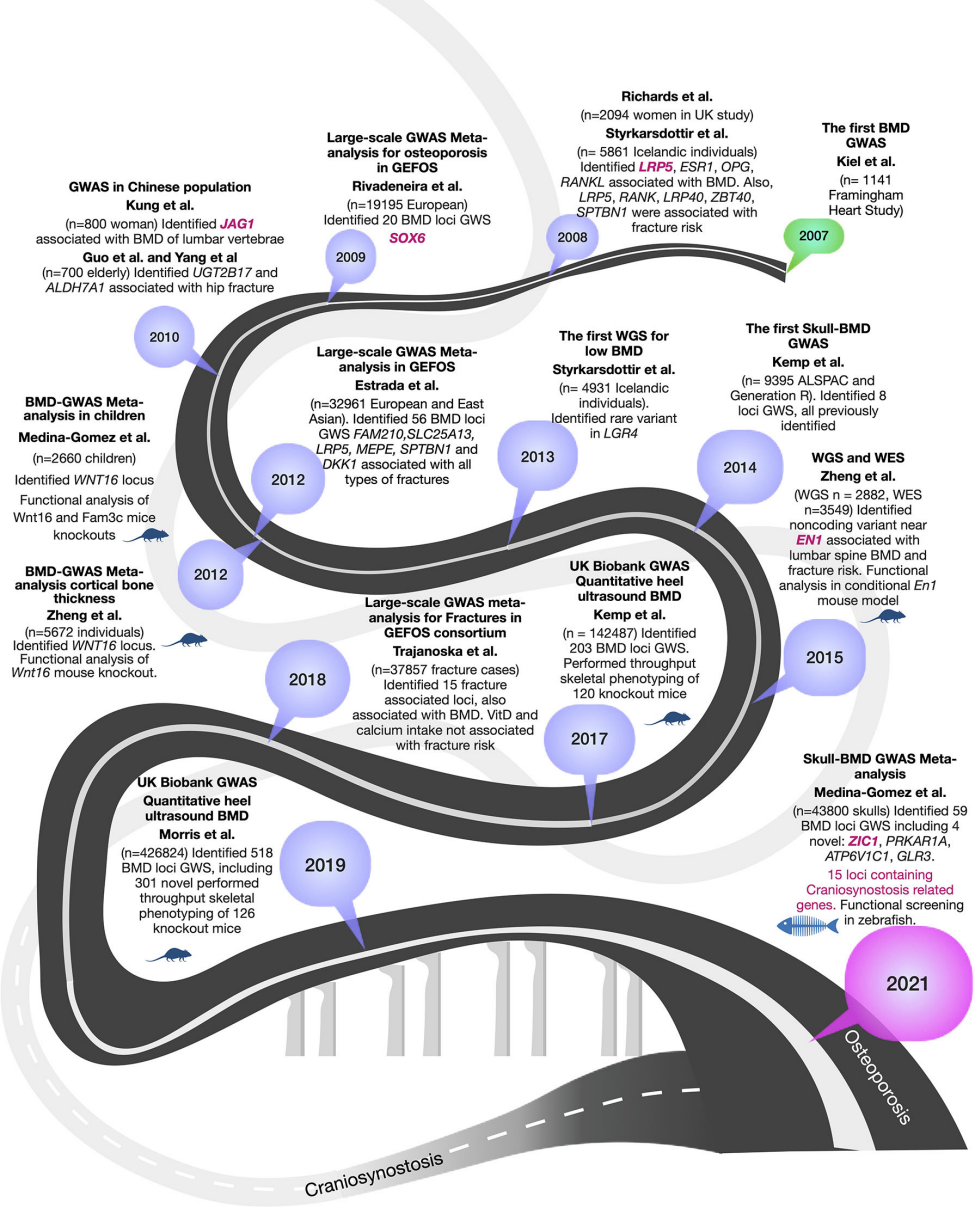
Summary of 15 years of GWASs for BMD to the current encounter with craniosynostosis. BMD-GWAS timeline is represented in a dark grey roadmap. Mouse and fish symbols indicate studies with functional work performed in mice and zebrafish, respectively. Genes highlighted in magenta are known craniosynostosis genes. A light grey roadmap represents the path by the genetics of craniosynostosis that finally meets with that of osteoporosis in 2021.

GWAS for osteoporosis mainly focused on investigating the effect of genetic factors on BMD, the strongest predictor of fracture risk; therefore this review will focus on BMD. Through the years, BMD GWAS have dramatically increased the number of participant and cohorts, resulting in the development of better methodologies for harmonizing data, expansion of reference panels for imputation, and cutting-edge tools for statistical analysis. More importantly, GWAS have progressively delineated genes to be prioritized for functional studies, so their biological function in the disease can be identified. In **Figure 1** we provide an overview of the GWAS for BMD.

The first BMD-GWAS was conducted in 2007 by Kiel et al. (19), including 1141 individuals from the Framingham Heart Study, but given the small sample size, none of the scrutinized SNPs reached genome-wide significance (GWS) (P< 5 x 10^-8^) (19). In 2008, two GWAS were published. Richards et al. (20) included 2094 women from the Twins UK study and carried out replication in 6463 participants from the Rotterdam Study (n=4081) and other European cohorts, identifying variants in *TNFRSF11B* and *LRP5* associated at GWS level (20). The second study, published by Styrkarsdottir et al. (21), was performed on 5861 Icelandic subjects and identified *TNFSF11* (*RANKL)*, *TNFRSF11B* (*OPG)*, *ESR1*, *ZBTB40*, and *TNFRSF11A* (*RANK)*. The authors also reported association of *SPTBN1*, *LRP4*, and *RANKL* with osteoporotic fractures (21). Since then, the greatest leaps in discoveries have evolved from extensive collaborative studies leading to the identification of hundreds of loci and SNPs associated with osteoporosis traits.

In 2009, Rivadeneira et al. (22) published the first large collaborative study of the Genetic Factors for Osteoporosis Consortium (GEFOS-1) cohort, including 19,195 European subjects. It identified 20 loci associated with BMD, of which 13 were novel (*GPR177, SPTBN1, CTNNB1, MEPE, MEF2C, STARD3NL, FLJ42280, LRP4. DCDC5, SOX6, FOXL1, HDAC5, CRHR1*). The meta-analysis also confirmed seven known BMD loci at GWS (*SBTB40, ESR1, TNFRSF11B, LRP5, SP7, TNFSF11, TNFSF11A*) (22). The second GEFOS study was published not long after three small GWAS in Chinese populations identified variants in *JAG1* (study of n= 800 women), *UGT2B17* (n=700 elderly, comprising 350 cases with a history of hip osteoporotic fracture and 350 healthy controls), and *ALDH7A1* (study of n= 700 elderly) (23–25), but the latter two were not replicated by subsequent studies. In 2012, the second large GEFOS-2 meta-analysis increased to 32,961 participants of Europeans and East Asia backgrounds (26). This study by Estrada et al. (26) identified 56 BMD GWS loci, including variants in 14 loci associated with all types of fractures (including *FAM210*, *SLC25A13*, *LRP5*, *MEPE*, *SPTBN1*, and *DKK1*) (26). In 2012, two other independent GWAS identified a prominent signal in *WNT16* (Wnt family member 16). Medina-Gomez et al. (27) analyzed 2,660 children of different ethnicities identifying *WNT16* locus influencing bone accrual (27). Zheng et al. (28) analyzed cortical bone thickness on 5,878 European subjects and identified an association with *WNT16*. Both groups validated their results with functional studies in homozygous mutant mice for *Wnt16-/-*, which resulted in 10% lower BMD, 27% thinner cortical bones at the femur midshaft and 43%-61% reduction of bone strength (28), therefore showing evidence of gene causality for this respective locus. Additional functional studies of *WNT16* locus were previously reviewed (29). Of note, rare coding variants in *CPED1* mapping in the same locus, appears to be also involved in regulating BMD variation (28).

With the advancements of next-generation sequencing technologies and prices for sequencing dropping down, it became realistic to move forward with the identification of rare variants (minor allele frequency, MAF<1%) through the incorporation of Whole-Genome (WGS) and Exome (WES) sequencing (reviewed by (29)). In 2013, the first WGS was performed by Styrkarsdottir et al. (30) for BMD in an Icelandic population comprising 4,931 individuals, which identifying a rare nonsense mutation (c.376C >T) within *LGR4* associated with low BMD and fracture risk (30). In 2015, Zheng et al. (31) used WGS [n = 2,882 from UK10K (sequenced 10K people to identify rare genetic variants in health and disease) (32)], WES, deep imputation of genotypes samples employing a combined UK10K/1000 Genomes reference (n=26,534), and *de novo* replication genotyping (n = 20,271). This effort resulted in the identification of a less frequent non-coding variant near a novel locus, harboring *EN1* (encoding a homeobox protein engrailed 1). Conditional mouse *En1* knockout presented reduced BMD and increased skull bone resorption (31). Further, non-coding variants mapping to *CPED1* were also identified, suggesting this gene in close vicinity of *WNT16*, is also involved in regulating BMD variation. In 2015, Styrkarsdottir et al. (17) performed GWAS using sequence variants found through WGS of 2,636 Icelanders, resulting in identifying two rare variants in the *COL1A2*, a gene known for to underlie many Osteogenesis Imperfecta cases, in association with low BMD and osteoporotic fractures. The carriers of these mutations did not show phenotypic signs of osteogenesis imperfecta but had low BMD (17). Collectively, these WGS-baswed studies underscore the importance of rare variants of larger effects in coding and non-coding regions that may not be identified through low powered GWAS. Despite the successful examples described here, the research community was already aware of the steep increase in sample size needed to adequately survey rare variation.

The emergence of large biobanks presented an unprecedented opportunity to assess the contribution of rare genetic variation to osteoporosis-related phenotypes within the so-called spectrum of “mega-GWAS” that gave rise to the two largest efforts in the osteoporosis field, studying estimated BMD (eBMD) measured with quantitative ultrasound of the heel. First, in 2017, Kemp et al. (15) undertook a GWAS in 142,487 individuals from the UK Biobank and identified 203 loci associated with eBMD, from which 153 were novel loci. This work used an exciting approach, among others, for gene prioritization. Genes within 1MB of any lead SNPs at the associated eBMD loci were checked for implication in knock-out mice models part of the International Mouse Phenotyping Consortium (IMPC) and the International Knockout Mouse Consortium (IKMC). In total, 120 genes had knockout mice available that presented abnormal bone structure after high-throughput skeletal phenotyping was undertaken by the Origins of Bone and Cartilage Disease (OBCD) consortium (15). In 2019, the largest GWAS to date on the genetics of osteoporosis was published as an extension of the eBMD analysis in the UK Biobank, involving now 426,824 participants (12). Morris et al. identified 515 loci, of which 301 were novel. This work involved skeletal phenotyping of 126 knockout mice, representing the largest GWAS with the largest functional evidence from mice.

This study dissected the contribution of independent lead SNPs per locus, showing that only 4.6% were rare (MAF<1%), whereas 9.3% were low frequency (1%>MAF ≤ 5%), and 86.1% were common (MAF>5%). Rare variants explained 0.8% of the variance in eBMD, whereas common variants explained 17.8%. Yet, it is important to mention that imputation of rare variants is challenging, and sequencing is essential to truly understand the impact of rare variations on human health. For instance, recently, the release of the UKBB exome sequencing revealed a catalog of 8-fold the coding variation accessible in the UKBB through imputation, in which a rare variant in *MEPE* was strongly associated with low BMD (33).

Beyond BMD, it is essential to mention the largest fracture risk GWAS performed by Trajanoska et al. (34). This GWAS on a set of 37,857 fracture cases and 227,116 controls resulted in the identification of 15 fracture-associated loci (*SPTBN1, CTNNB1, RSPO3, ESR1, WNT16/CPED1, SHFM1, STRARD3NL, GRB10/COLBL, FUBP3, MBL2/DKK1, LRP5, RPS6KA5, SOST, FAM210A, ETS2*) from which all had been previously associated with BMD. Through two-sample Mendelian randomization, the author assessed different risk factors for fracture; BMD and hand grip strength showed significant evidence for a causal effect; whereas genetically-determined vitamin D levels and calcium intake were not associated with fracture risk. Other relevant GWASs were also published during the mentioned period (35–38).

To date, GWAS for BMD have significantly advanced our knowledge of the complex genetics underpinning osteoporosis, shed light on the genetic causes of fracture risk, and hinted on the molecular mechanisms underlying bone homeostasis. BMD measurements constitute excellent endophenotypes of fracture risk, while also pinpointing diverse aspects of bone biology and homeostasis. For example, significant genetic correlation has been described between BMD loci and those of bone growth and shape (39), osteoarthritis (40, 41), high bone mass disorders (42), rare skeletal disorders (17), Paget’s disease of bone (16, 43), metabolic disorders (i.e., diabetes) (44), etc. Such genetic overlaps indicate that diverse factors and pathways regulate the skeletal mineral content in our bodies. By studying bone conditions of known genetic causes and dissecting their function to maintain BMD, we will learn key elements in bone biology with the potential of translating this knowledge into therapeutic strategies for bone diseases.

Recently, a curious overlap between BMD and craniosynostosis, a condition that affects the cranial bones, has emerged. We may ask ourselves, “what does the skull have to do with osteoporosis?”

## Why should we give attention to the skull?

While most GWASs were performed using BMD at the femoral neck and lumbar spine, less attention has been given to the skull. However, the skull has a few attributes that may allow the identification of new genetic factors in bone biology and, surprisingly, also for osteoporosis.

The skull (not including the jaw) is subjected to considerably less mechanical loading than axial or appendicular bones. Studies in mice estimated that the human fibula has a load nearly twice that of the skull bones (45). *In vivo* studies in humans showed that parietal bones in the skull were subjected to at least ten times lower loads than the appendicular skeleton (46). The skull is subject to a distinctive mechanical forces pattern, loaded radially and tangentially by intracranial pressure and mastication (45). This is also reflected in the morphology of mechanosensing osteocytes in the skull compared to those present in the tibia, as demonstrated by studies in mice. Skull bones have fewer osteocytes with smaller bodies but increased surface area, due to the increased number of cellular processes (47, 48). Skull of rats highly express genes in response to mechanical loading in the limbs (48). These findings suggest distinct molecular adaptations of mechanosensing osteocytes in the skull, which can maintain bone mass despite being subjected to lower mechanical loading than appendicular bones.

Interestingly, the calvaria does not lose bone during disuse or after menopause (49). In contrast to the rest of the body, cranial bone mass does not decline during spaceflight (50). Similarly, prolonged bed rest and spinal cord injury result in bone loss from the appendicular and axial skeleton but not from the skull (51, 52). Therefore, GWAS derived from skull BMD may represent an excellent source for identifying genes associated with yet to be elucidated mechanosensing properties and subjected to fewer environmental influences (i.e., physical activity) than appendicular bones.

Another peculiarity of the skull is its embryonic origin and bone formation modes. The skull is formed from cranial skeletogenic mesenchyme derived from two distinct embryonic sources: mesoderm and neural crest (53). The neural crest cells are multipotent stem cells that, in early development, arise in the dorsal-most aspect of the forming neural tube, from which undergoes an epithelial-to-mesenchymal transition to delaminate from the neuroepithelium. Neural crest cells migrate extensively throughout the early embryo to contribute to important cell types, including the peripheral nervous system, the craniofacial skeleton, and the cardiovascular system (54). Lineage tracing studies in mice, chicken, and zebrafish have demonstrated that the frontal bones are derived from neural crests, while parietal bones originate from the mesoderm (53, 55, 56). Macroscopically, at birth, the skull has movable fibrous regions, providing plasticity required during delivery and later for the growth and development of the head and brain. These regions are composed of dense connective tissue shaped into six fontanelles: anterior (frontal), posterior (occipital), anterolateral (sphenoid 2x) and posterolateral (mastoid 2x); which fuse normally by the age of 12 to 18 months. Although 80% of skull adult size is reached by the age of 3, bone growth continues *via* membranous ossification.

When it comes to types of ossification, the skull is complex as it is formed both by intramembranous (without a cartilaginous intermediate as seen in the calvaria) and endochondral ossification (temporal and skull-base). Mesenchymal cells within a vascularized area of the connective tissue proliferate and differentiate directly into preosteoblasts and finally into osteoblasts (57). The structure of the flat bones of the skull resembles a sandwich, where cortical bones represent the bread, while the center of the sandwich is the cancellous (trabecular) bone. The amount of cortical bone in the skull exceeds that of trabecular bone. Hence, GWAS on the BMD of the skull may primarily highlight genes associated with cortical bones and highly mechanosensing.

Finally, the skull is often compromised in monogenic skeletal conditions, exemplified by patients with recessive mutations in *SOST* (encodes sclerostin). In both cases of *SOST*-related sclerosing bone dysplasia, including sclerosteosis and van Buchem disease, patients display excessive bone formation (hyperostosis) phenotypically, leading to increased bone mass throughout the body, but particularly thickening of the skull bones (58, 59). *SOST* codifies sclerostin, a secreted glycoprotein predominantly produced by osteocytes and known to inhibit bone formation (60). Remarkably, the identification of *SOST* led to the development of romosozumab. Although with concerns of cardiovascular complications, this humanized monoclonal antibody inhibits sclerostin and is currently the most effective drug for osteoporosis, approved in Europe in 2019 (61). Calvaria thickening is also observed in cases of pyknodysostosis, a rare autosomal recessive bone dysplasia caused by mutations in *CTSK* (cathepsin K), characterized by osteosclerosis and short stature. Cathepsin K is among the most attractive targets for anti-osteoporosis drug development, reducing resorption while maintaining bone formation. Cathepsin K inhibitor odanacatib demonstrated high therapeutic efficacy in patients with postmenopausal osteoporosis in Phase III clinical trials. Still, unfortunately, due to cardio-cerebrovascular adverse effects, it was not approved to go into the market (62). More examples will be highlighted throughout the text. Still, it is essential to notice that genetic conditions affecting skull intramembranous bones have been vital in developing potent therapeutics for osteoporosis.

## Craniosynostosis is the premature closure of the sutures between the flat bones of the skull

Craniosynostosis is a major structural birth defect characterized by the premature fusion of one or more cranial sutures (18). It happens in 1 in 2,500 liveborn babies, with approximately 80% of children presenting an isolated phenotype (i.e., nonsyndromic craniosynostosis), with the suture fusion being the only defect (63). The remainder of children with craniosynostosis present with syndromic craniosynostosis that includes additional birth defects and developmental delays (64).

**Figure 2:**
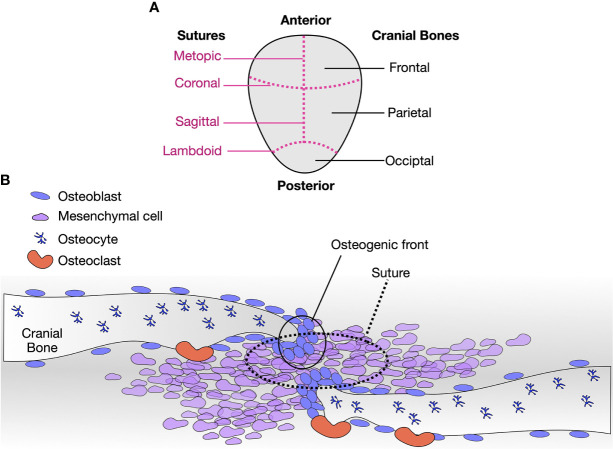
Cranial sutures are paradigms to study osteoblast differentiation, remodeling, and regeneration. **(A)** Diagram of a human calvaria, with respective cranial bones (right, black) and sutures (left, magenta, dashed lines). **(B)** Diagram of a suture. Osteoblasts, osteocytes, osteoclasts, and mesenchymal cells are indicated. The tip of each cranial plate is a region of active osteoblasts bone deposits. Osteoblasts lining cells are positioned away from the fronts and along the bone. Osteocytes are embedded in the bone matrix, and osteoclasts resorb bone. At the sutures, pluripotent mesenchymal cells self-renew and eventually enter osteoblastic differentiation to contribute to the osteogenic fronts. As observed with craniosynostosis, minor molecular disturbances at the sutures lead to severe phenotypes.

The cranial sutures bridge the gap between the cranial bones, allowing to mold the head while passing through the birth canal and are growth sites of the skull. At the suture osteogenic fronts, a balance between proliferation and differentiation ensures the coordinated growth of the calvaria during the first years of life (64) (**Figure 2**). Within the sutures, a stem cell skeletogenic niche is found, self-renewing and allowing bone modeling, remodeling, regeneration, and healing over an extensive period during the skeletal development (65) (**Figure 2**). The metopic sutures, located between the frontal bones, are the first to close at approximately nine months of age. The fusion of the remaining sutures are largely completed by 18 months, but complete fusion does not occur until the third decade of life (64). Therefore, the sutures compromised in craniosynostosis comprise a unique paradigm for studying osteoblast differentiation, bone remodeling, and regeneration.

In the presence of premature closure of the cranial sutures, the brain cannot expand naturally during growth. Thus, affected children may experience ongoing medical complications, such as increased intracranial pressure (66, 67), vision problems (optic neuropathy secondary to elevated intracranial pressure, strabismus, refracture errors, exposure keratopathy from exorbitism) (68), and learning disabilities (69–71). As consequence, children with craniosynostosis require extensive surgical treatment through their first years of life to correct the malformation and allow brain expansion. However, after correction surgeries, re-synostosis is a recurrent problem requiring follow-up surgeries, given the enhanced osteoanabolic activity in the cranial sutures (72). The complexity and number of surgeries needed reflect the healthcare and financial burden caused by craniosynostosis. Some data exist regarding the long-term results of the surgical corrections (73, 74) and developmental outcomes (75, 76).

Despite the high healthcare burden of craniosynostosis, the genetics of craniosynostosis started to be elucidated only in 1993, when Jabs et al. identified a mutation in *MSX2* in a patient with Boston-type syndromic craniosynostosis, marking the first gene discovered as causing craniosynostosis (77). Since then, the genetic causes of most syndromic cases have been identified (78, 79). These include mutations in *FGFR2* explaining Apert, Crouzon, Pfeiffer, and Jackson-Weiss syndromes; mutations in *FGFR3* leading to Muenke syndrome and acanthosis nigricans, in *TWIST1*, causing Saethre-Chotzen syndrome; mutations in *JAG1* leading to Alagille syndrome, *ERF*, *TCF12* causing *ERF*-related and *TCF12*-related craniosynostosis, and other mutations associated with Mendelian forms of syndromic craniosynostosis (64, 80–82). Nearly 100 genes have been identified as causative of craniosynostosis (79). Syndromic cases have also been associated with several chromosomal aberrations (78).

Approximately 80% of craniosynostosis cases are non-syndromic and of unknown genetic cause (79, 83, 84). Most children with nonsyndromic craniosynostosis present as sporadic cases, although an estimated 6-8% of families have multiple affected individuals (85). Segregation analyses of multiplex nonsyndromic craniosynostosis families support an autosomal dominant inheritance with 38% penetrance for sagittal nonsyndromic craniosynostosis (86) and 60% penetrance for coronal nonsyndromic craniosynostosis (87), although pedigrees suggestive of autosomal recessive inheritance have also been observed. The etiopathogenesis of nonsyndromic craniosynostosis represents a significant gap in our current knowledge, but genomic technologies have begun to narrow this knowledge gap. In 2012, Justice et al. (88) performed the first GWAS for sagittal nonsyndromic craniosynostosis, using 130 non-Hispanic case-parent trios of European ancestry (88). This study identified novel, strong associations to *BMP2* and *BBS9* (88). Functional studies using zebrafish demonstrated that rs1884302 in the vicinity of *BMP2* acts as an enhancer (89). In 2020, Justice et al. (90) published the second GWAS of nonsyndromic metopic craniosynostosis using 225 non-Hispanic case-parent trios, identifying six GWS SNPs: rs781716 intronic to *SPRY3*, rs6127972 intronic to *BMP7*, rs62590971 ~155 kb upstream from *TGIF2LX*, and rs2522623, rs2573826, and rs2754857 all intronic to *PCDH11X* (90). A replication study using an independent cohort of 194 unrelated metopic cases and 333 unaffected controls, confirmed rs6127972 mapping nearby a member of the bone morphogenetic protein (BMP) family *BMP7.* Furthermore, WES studies have identified *SMAD6* loss-of-function mutations in 7% of midline nonsyndromic patients (91), mutations in *ERF* in 5 of 12 nonsyndromic multiplex families (92), and mutations in *TCF12* in 10% of unilateral coronal nonsyndromic craniosynostosis and 32% of bicoronal cases (93). More complex presentations are well-characterized by the discovery of a two-loci inheritance resulting in midline craniosynostosis, involving interaction between rare coding variants in *SMAD6* and the risk allele of rs1884302 in the vicinity of *BMP2*, finding that has been replicated (94).

Human fused suture specimens, although important, are a limited resource that does not allow for comprehensive investigations of events leading to suture fusion. Thus, animal models remain critical for investigating the alterations within cranial sutures that precede fusion. Traditionally, mice have been used to study craniofacial development and become fundamental to our current understanding of craniosynostosis. Mouse models have been developed for syndromic forms of craniosynostosis, such as Apert, Crouzon, Muenke, and Saethre–Chotzen syndromes (95, 96), and a limited number for nonsyndromic craniosynostosis (97–100). These models have served as a proof-of-principle for nonsurgical craniosynostosis treatment, as observed from prenatal phenotype rescue of Apert and Crouzon mouse models with small molecules (101, 102) and of midline nonsyndromic craniosynostosis with rapamycin (103). However, these mouse models do not allow visualization of suture formation *in vivo* and do not serve as rapid screening systems for testing genes in loci identified by GWAS, WGS and/or WES.

Children diagnosed with craniosynostosis are rarely systematically evaluated longitudinally for late-onset morbidities, limiting our knowledge about the late effects mutations in craniosynostosis genes have on our entire skeleton. However, there is evidence that some craniosynostosis genes also regulate whole-body bone homeostasis, such as mutations in *LRP5* causing osteoporosis-Pseudoglioma, *ALPL* causing low serum alkaline phosphatase, and low-BMD, *PHEX* leading to hypophosphatemia rickets and diminishing bone regenerative capacity (79), etc. Moreover, all known craniosynostosis causative genes are also identified by GWAS for human height (msk.hugeamp.org), suggesting the role of bone growth in the etiology of the condition.

## Osteoporosis meets craniosynostosis

In 2014, Kemp et al. (104) published the first GWAS for skull BMD (SK-BMD) in pediatric populations. First, the study estimated the heritability of BMD across skeletal sites. For this, they partitioned total body-BMD into lower-limb, upper-limb, and skull using total-body DXA scans of 4,890 participants recruited by the Avon Longitudinal Study of Parents and their Children (ALSPAC). Interestingly, common SNPs explained a greater proportion of the overall variance of SK-BMD when compared to BMD measured at the appendicular sites. Such difference might reflect the differential exposure of each skeletal site to environmental influences, particularly those acting through the mechanical load, suggesting that skull BMD is less influenced by environmental factors. Next, combining data from ALSPAC with the Generation R Study, they performed a GWAS meta-analysis on 9,395 participants, identifying eight loci associated with SK-BMD, all of which were previously associated with BMD at appendicular regions, suggesting distinct architecture of gene regulation among skeletal regions (104). These regions included *TNFR11A1*, *TNFS11*, *RSPO3/CENPW*, *WNT16/CPED1*, and *WNT4*, all genes with essential functions in bone biology and previously associated with osteoporosis. For example, *RSPO3* is one of the top genetic determinants for fractures and has been robustly associated with increased BMD (12, 34). Later, functional studies demonstrated *RSPO3* expression in osteoblasts, blood cells, adipocytes, and skeletal stem cells (105). *RSPO3* expression leads to increased osteoblast proliferation and differentiation. Conditional mice *Rspo3* knockouts showed that osteoblasts are the principal source of its expression in bone and a central regulator of trabecular bone mass (105). Lead SNPs in the *RSPO3* locus associated with SK-BMD differ from those identified associated at the lumbar spine and femoral neck, pointing to different regulatory elements governing the gene expression across skeletal sites (104). This study suggested that mostly the same genes would be identified from SK-BMD GWASs, but with variations in lead SNPs, and gene regulation.

Recently, we conducted the largest GWAS for SK-BMD (106), including 43,800 participants. By increasing the sample size from the previous work, we identified 59 loci, of which 4 were novel (*ZIC1*, *ATP6V1C1*, *PRKAR1A*, and *GLRX3*). Indeed, our study demonstrated that most genes associated with SK-BMD also partake in axial skeletal homeostasis, as fifty-five out of fifty-nine SK-BMD loci (93%) co-localized with total-body-BMD loci. *RSPO3* locus also displayed independent lead SNP, as previously identified by Kemp et al. (104). We highlighted associated variants mapping to known BMD genes that belong to 1% of most deleterious skeletal variants in the human genome (*e.g., LRP5* (107), *WNT16* (108), *LGR4* (109)*, CPED1* (27)*, LRP4* (110)). Association with *SOST*, which provides the genetic basis of romosozumab, a recently developed osteoporosis medication (61), was also detected. Our skull-BMD study underscored genes with mechanosensing properties, including the novel BMD-associated gene *ZIC1*, which encodes a zinc-finger transcription factor. A few studies have demonstrated mechano-function properties of *ZIC1*, including its differential expression in skeletal regions of higher and lower mechanical load, also *ZIC1* immunolocalization in the cytoplasm, and translocation to the nucleus of murine osteocytes upon fluid shear and stress experiments (111, 112). While more studies are needed to evaluate *ZIC1* function in osteocytes and its function as a mechanosensing gene, its current understanding corroborates the high potential of GWAS for SK-BMD to identify genes with mechanosensing properties.

Additionally, our GWAS for SK-BMD revealed a remarkable overlap between BMD loci and genes associated with craniosynostosis. Fifteen of the 59 SK-BMD loci (25%) harbored craniosynostosis causative genes or family members of causative genes (**Figure 1**). Among known craniosynostosis causative genes were *EN1*, *IDUA*, *CSNK1G3*, *DLX6*, *SOX6*, *JAG1*, *LRP5 (* 79), and the novel candidate BMD gene *ZIC1 (* 84). Mutations in craniosynostosis genes lead to premature suture fusion frequently due to enhanced osteoblast activity or reduction of the mesenchymal cell pool (64). Because the sutures experience a very fine regulation of osteoblast differentiation, small changes in this process lead to severe craniofacial phenotypes. This implies that the genetic overlap between BMD and craniosynostosis automatically flags the gene’s function partaking in osteogenesis and possibly with osteoanabolic properties. By studying the molecular mechanisms of suture fusion, we may learn shortcuts to decipher a gene’s biological impact on bone homeostasis of other parts of the body. For example, at the sutures, *EN1* influences osteoblasts, and it has a non-cell-autonomous function in regulating osteoclast recruitment and activation, affecting the resorption and remodeling, as demonstrated by studies in mice (113). *EN1* has been robustly associated with BMD at different skeletal sites and fracture risk (31). Additional functional studies in mice showed *En1* expression in osteoblast cells of cortical and of lumbar trabecular bone and in osteocytes of cortical bones. Conditional mice knockout showed lower trabecular bone volume, number, and thickness, reduced femoral cortical thickness and increased osteoclast activity (31). Therefore, this points to similar mechanisms in osteogenesis as in suture fusion. *En1* is expressed in the paraxial cephalic mesoderm, forming an important cell population above the mice developing eye that migrate to form the coronal suture (114). *Zic1* is expressed in the same cellular domain as *En1* (84) and is homologous to the *Drosophila* gene *odd-parred*, which is required to activate embryonic *engrailed* expression (115). The novel BMD gene, *ZIC1*, may regulate *EN1* in bone development and homeostasis. Gain-of-function mutations in *ZIC1* cause coronal craniosynostosis and learning disability. In mice, *Zic1-/-* display cerebellar hypoplasia and vertebral fusions, similar to Gli3 knockout (Gli-Kruppel family member 3), which is highly important for mesenchymal cell patency in the sutures (116, 117). The joint involvement of *ZIC1*, *EN1*, and *GLI3* during bone formation and homeostasis has yet to be determined.

The association of noncoding variants in the 3’ region of *BMP2* resulting in nonsyndromic sagittal craniosynostosis points to another interesting intersection with osteoporosis. Craniosynostosis occurs due to accelerated osteoblast differentiation with increased BMD implying overexpression of *BMP2*. Since osteoporosis involves opposite processes (i.e., decreased osteoblast differentiation and activity), the mechanisms leading to low BMD would involve downregulation of *BMP2*. Indeed, Styrkarsdottir et al. (118), reported three *BMP2* variants, a missense polymorphism, and two anonymous single nucleotide polymorphism haplotypes, to be associated with osteoporosis in the Icelandic patients (118).

Because the sutures are a source of pluripotent mesenchymal cells with regenerative capacity (119), identified BMD genes that are expressed in the suture mesenchyme and involved in craniosynostosis may be exciting targets as osteoinductive in bone regeneration. This is supported by the Notch ligand *JAG1*, where mutations in the gene lead to Alagille syndrome and craniosynostosis, and was one of the first genes found associated with BMD and osteoporotic fractures by GWAS in 2010 (23, 82). Studies in mice have shown that *Jag1* is expressed in the suture mesenchyme, functioning along *Twist1* in maintaining suture patency, and downregulation of *Jag1* leads to enhanced osteoblast differentiation (82). Mice studies also showed that *Jag1* is required for normal trabecular bone formation, leading to downregulation of osteoblast differentiation genes in trabecular bone and osteopenic phenotype (120). Remarkably, Jag1 has emerged as a potent osteoinductive protein that positively regulates post-traumatic bone healing in the last decades. Intraoperative delivery of Jag1 with collagen sponges at the fractures has rendered exceptional and promising healing results without ectopic bone formation, observed with BMPs (121). This bond opens opportunities to explore the osteoinductive potential of other craniosynostosis genes for therapeutic approaches in fracture healing.

Nevertheless, several questions remain unanswered, such as the primordial question of whether craniosynostosis genes are indeed causal in BMD, or whether skull BMD genes are involved in skull development and/or craniosynostosis. For this, functional studies in animal models that allow a longitudinal analysis of skull formation and skeletal homeostasis will provide valuable insight.

## Zebrafish for functional studies of craniosynostosis and osteoporosis genetics

Mice have been the traditional animal models for functional experiments in the osteoporosis and craniosynostosis fields (15, 84, 92, 105). However, zebrafish (*Danio rerio*) have attracted recent interest in the functional validation of genetic findings from human studies. But not only that, they are becoming preferable models for higher throughput rapid phenotypic and genetic screening, especially for the scrutiny of genes identified by GWAS. Zebrafish are freshwater fish of relatively simple maintenance and low cost, of external fertilization and rapid development, that weekly generates over 200 individuals per cross (122). The zebrafish community has made available an arsenal of genetic tools (i.e., reporter lines, site-directed mutagenesis, reverse and forward genetic screenings, etc.) and phenotypic tools for skeletal studies in zebrafish (123, 124). Despite zebrafish having evolutionarily diverged from humans around 450 million years ago, about 80% of disease-causing genes in humans have an ortholog in zebrafish (125). During evolution, zebrafish passed through additional whole-genome duplication, which resulted in gene duplicates (orthologs to mouse/human genes). In many cases, orthologs functionally balance each other, permitting to phenotypically study of individual orthologs in zebrafish that are embryonic lethal in mouse (126, 127). Also, while mutations in many skeletal genes are lethal in mice, zebrafish carrying mutations in the same genes may survive to adulthood, due to the supportive buoyancy in the aquatic environment (128). The molecular pathways and types of ossification (endochondral and intramembranous) are conserved between zebrafish and humans, with zebrafish displaying osteoblasts, osteoclasts, osteocytes, and chondrocytes (129, 130). For these many reasons, zebrafish have contributed to an increasing number of models for human skeletal diseases over the years (reviewed by Kague et al. (29)).

**Figure 3:**
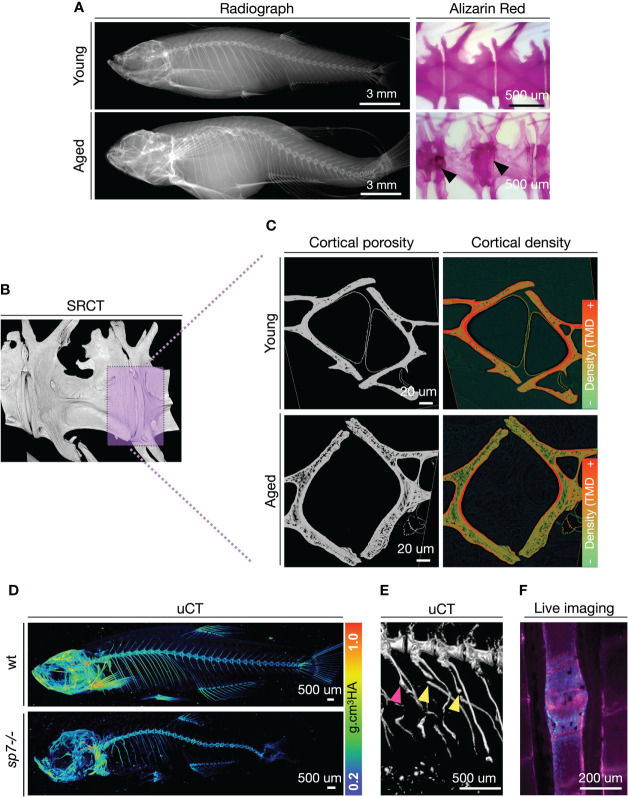
Zebrafish develop bone microarchitecture deterioration during aging, and young zebrafish mutants are used to study osteoporosis-associated genes. **(A)** Radiographs and Alizarin Red Staining of young (1 year = ~30 human years) and aged (3 years = ~80 human years). Alizarin red staining from Kague et al. (123). Note bone sclerosis in endplates of aged spines (arrowhead). **(B)** Synchrotron radiation µCT of a region of interest in the zebrafish spine. The magenta box shows a selected region for a digital cross-section. **(C)** Digital cross-sections of the endplate region of the zebrafish vertebral column to show cortical porosity (greyscale) and density (colored by pixel grey intensity). TMD, Tissue Mineral Density. Regions of higher densities are shown in red, while lower densities are in green. **(D)** Micro-computed tomography (µCT) of young (3 months old) wild-type (wt) and *sp7-/-* mutant with values of tissue mineral density (cortical density). µCT images from Kague et al. (123). **(E)** Fractures are observed in ribs of *sp7-/-* mutants. Fractures (yellow arrowhead), calluses (magenta arrowhead). **(F)** Example of a fracture in the zebrafish caudal fin imaged from live fish. Calcified bone was stained with Alizarin Red (magenta) and osteoblasts (blue) are labeled with Tg(*sp7:gfp*) reporter line. Scale bar is represented in each image.

Adult zebrafish models to study osteoporosis have only been developed recently, alongside studies characterizing skeletal changes during aging (123, 131, 132). Recently, we provided strong support for osteoporosis in aging zebrafish, showing that aged zebrafish spines display increased susceptibility to fractures and have bone quality deterioration (increased cortical porosity and tendency towards reduction of BMD), reminiscent of the osteoporosis (**Figures 3A–C**) (123). While aging zebrafish studies allow us to learn how to recognize critical hallmarks of skeletal architecture deterioration, varying from micro to nanoscale, under genetic manipulation, young zebrafish provide consistent and compelling models for osteoporosis. Mutant zebrafish are attractive models for functional studies referent to adult skeletal maintenance, with BMD changes easily detected by standard techniques (**Figure 3D**). This has been demonstrated with several mutants, including zebrafish *sp7-/-* and *lrp5-/-* among others (123, 133). By using high-resolution 3D imaging (<0.1um, synchrotron radiation), osteocyte lacunar profile can also be obtained (123, 134).

While tools for the prediction of fractures risk in zebrafish have not been developed yet, genetically modified zebrafish offer alternative ways to study fracture occurrence, prediction, and fracture healing (135, 136). Due to buoyance in the aquatic environment, fish are subjected to a lower mechanical load than terrestrial species. However, swimming behavior through a viscous medium plays an important factor in mechanical resistance to the zebrafish skeleton. This is confirmed by recent findings of vertebral compression fractures in zebrafish modeling osteogenesis imperfecta (137). Also, zebrafish subjected to an exercise regime increase bone formation at the vertebral endplates (138), while those subjected to restricted mobility develop osteopenia (139). Fragility fractures are often observed in ribs and fins (**Figures 3E, F**) (128, 140). The zebrafish bony fin rays, known by fish experts as lepidotrichia, are regions of interest to monitor bone fractures. Aged zebrafish show increased numbers of spontaneous fin fractures, detected by the presence of calluses (**Figure 3F**) (141). Calluses are also observed prematurely in young zebrafish carrying a loss-of-function mutation in *wnt16* or *bmp1a1* (136, 141). Moreover, a fracture can be induced by applying pressure on fish fins, and fracture healing can be monitored longitudinally *in vivo*.

**Figure 4:**
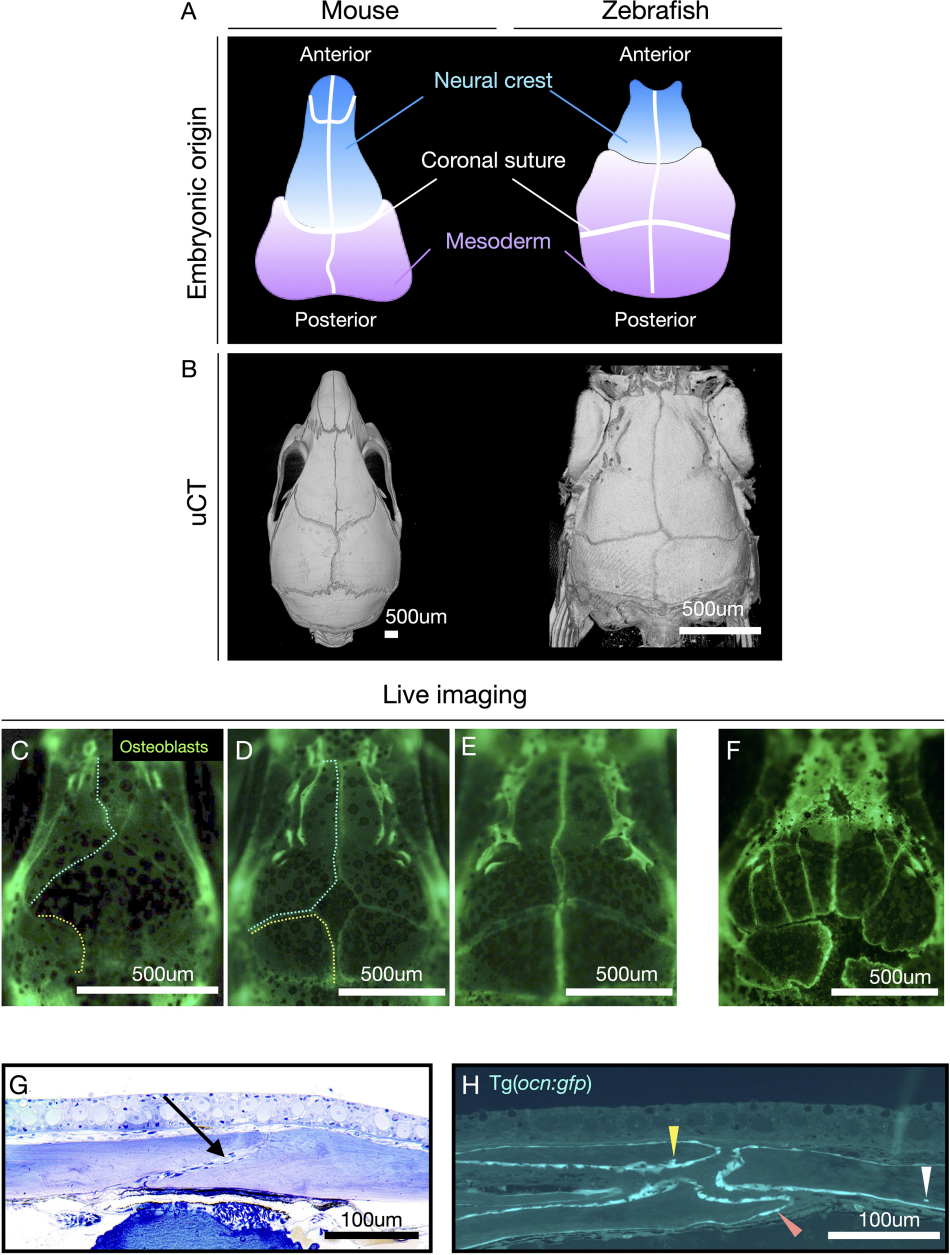
Zebrafish have the same sutures as mammals with the advantage of allowing the visualization of suture formation *in vivo*. **(A)** Diagram showing the contribution of neural crest (blue) and mesoderm (lilac) cells to the mouse and zebrafish calvariae (top panel). Note: coronal suture in zebrafish is formed by mesoderm/mesoderm while in mice by neural crest/mesoderm. **(B)** µCT of calvaria of mice and zebrafish. **(C–E)** Live imaging of wild-type (wt) zebrafish calvariae during suture formation using Tg(*Runx2:egfp*) reporter line labeling osteoblasts. Note: osteogenic fronts of the frontal bones (cyan dashed line) and the parietal bones (yellow dashed line). Bone growth is initiated from lateral condensations and progress towards the middle of the calvaria to totally cover the brain. **(F)** Live imaging of *sp7-/-* as an example of abnormal suture patterning, and ectopic sutures. **(G)** Histological semi-thin section of adult male zebrafish, at the coronal suture, stained with Toluidine Blue. Note: in contrast to humans bones overlap at the suture. The coronal suture is indicated with an arrow. **(H)** Semi-thin plastic section at the sagittal suture of a 3-month-old zebrafish carrying the reporter line Tg(*ocn:gfp*) labeling osteoblasts and osteocytes (expressing osteocalcin) (according to protocol (144)). Osteoblasts (orange arrowhead), terminal osteoblasts being immersed in bone matrix (yellow arrowhead), osteocyte embedded in bone (white arrowhead). Images **(G, H)** were kindly provided by the laboratory of Dr. P. Eckhard Witten. Scale bars are indicated in each image.

One of the fascinating advantages of zebrafish is their transparency, which prevails from early development to the formation of the main skeleton during juvenile stages. This permits *in vivo* cell traceability while the skeleton is formed using reporter lines labeling specific cell types (i.e., osteoblasts, osteoclasts, and chondrocytes) (14, 126). Zebrafish cranial bones and sutures are homologous to those in mammals, representing the only vertebrates enabling non-invasive *in vivo* visualization of cranial bones growth and suture formation (55, 128), therefore of great interest for models of craniosynostosis (89, 142, 143). Zebrafish develop the same sutures as humans and mice (55, 128) (**Figures 4A, B**). In 2012, we mapped the contribution of neural crest cells to the whole zebrafish adult skeleton and the skull, taking advantage of the cre-lox system, where cre was expressed in neural crest cells through an enhancer for *sox10* and used to permanently label neural crest derivatives (55). We demonstrated that the anterior part of the frontal bones was neural crest-derived and the remaining posterior part, including the coronal suture, was of mesoderm origin (55). Therefore, while the coronal suture in mice is of neural crest-mesoderm origin, our experiments demonstrated that the coronal suture is formed by mesoderm-mesoderm origin in zebrafish.

Additionally, the zebrafish sutures do not fuse during their lifetime, and the two cranial flat bones remain overlapping (**Figures 4A, G, H**), highlighting a difference from humans (128). However, these differences are insufficient to prevent craniosynostosis in zebrafish genetic models. Craniosynostosis was first modeled in zebrafish through loss-of-function mutations in *cyp26b1*, reproducing coronal craniosynostosis observed in human patients (143). Later, Teng et al. (142) demonstrated that bi-coronal synostosis could be reproduced in zebrafish when *twist1b* and *tcf12* were simultaneously knockout, similarly to results in mice knockouts (142). The group also showed that mutations in these genes led to the reduction of osteoprogenitor cells at the suture mesenchyme culminating in the coronal synostosis (142).

Advantageously, given the transparency of zebrafish and the superficial position of the cranial bones, the sutures can be studied longitudinally as they are formed (**Figures 4C–F**). We were the first to show *in vivo* formation of the calvaria and the formation of ectopic sutures in zebrafish lacking the important osteogenesis gene *sp7* (**Figure 4F**) (128). By using transgenic lines labeling osteoblasts, it is simple to detect the osteogenic fronts of the sutures, sites of dynamic bone growth, and renewal (**Figures 4C–F, H**). Genes associated with maintenance of suture pattern when mutated in zebrafish often result in the formation of ectopic sutures, as seen in *sp7* (128)*, twist1a, twist1b* (142)*, fgfr3 (* 145), and *bmp7* (unpublished data). Another peculiarity of mutations in craniosynostosis genes in zebrafish is their association with morphological changes in suture position and differences in calvariae bone growth rates (142, 145). The ossification centers, formed laterally in the skull next to the eyes, as in mice, are essential regions of mesenchymal condensation and initial bone formation. The genes associated with these steps in osteogenesis timely regulate bone condensation and growth rate. Delays during condensation lead to fontanelles, ectopic bones, and changes in suture pattern (**Figure 4F**) (128). These phenotypic changes serve as hallmarks indicating a gene’s function in suture homeostasis and bone growth. Therefore, they can be easily detected during phenotypic screenings.

Remarkably, zebrafish models have become of great interest to pinpoint causal genes in GWAS loci. The main reason for its suitability is the unbeatable speed to test individual or multiple genes in zebrafish. The CRISPR/Cas9 technology efficiently operates in zebrafish, permitting a reliable recapitulation of complex mutant phenotypes in the first generation (mosaic G0s, commonly known as crispants), as demonstrated for genes associated with osteogenesis imperfecta and osteoporosis (124, 133). Crispants involved with osteogenesis imperfecta (*PLOD2*) and osteoporosis (*LRP5*) were able to reproduce similar phenotypic characteristics, such as low BMD, like those of knockout mutants (124, 133). Zebrafish crispants represent powerful tools for rapid functional annotation of GWAS-identified variants, able to add a new layer for gene prioritization based on animal findings. Altogether, among the advantages that zebrafish models can offer to the functional evaluation of genetic associations, they represent the ultimate optimum to study the genetic overlap between osteoporosis and craniosynostosis.

Our SK-BMD is the first study that has combined adult zebrafish to provide functional support for GWAS findings in BMD (106). Taking advantage of crispants and the transparency of zebrafish, we tested selected genes harbored in novel identified BMD loci, *ZIC1*, *ATP6V1C1*, and *PRKAR1A*. These genes were targeted by CRISPR/Cas9 to cause loss-of-function mutations and analyzed longitudinally under a fluorescent microscope to visualize cranial suture formation *in vivo.* Invariably, we detected suture mis-patterning, ectopic sutures, and abnormal bone growth associated with *zic1* crispants. Similar results were found for *atp6v1c1;* while for *prkar1a*, only abnormal calvarial bone growth was detected. These results suggest that *ATP6V1C1* is a BMD-associated gene of potential interest as causative of craniosynostosis and should be further investigated in unresolved forms of the condition. Moreover, micro-computed tomography revealed BMD changes supporting the role of these three genes in bone homeostasis and control of bone mass. These results constitute an excellent starting point, exemplifying the great potential that SK-BMD GWAS and functional validation in zebrafish will bring to the fields of osteoporosis and craniosynostosis.

## Future perspectives

The genetic overlap between osteoporosis and craniosynostosis only became apparent recently through the GWAS for SK-BMD. However, this intersection actually dates back to the first successful GWAS in 2008 reporting variants mapping to *LRP5 (* 21), then in 2010 *JAG1* was reported (23), and more recently, the discovery of *EN1* in 2015 (31) (**Figure 1**). Whether this overlap is merely a coincidence is unlikely. Some craniosynostosis genes are essential for the critical balance between bone formation and resorption, a process that is mirrored by measurements of BMD (64, 79). One can conclude that the pool of genes involved in overall bone function is very similar across distinct skeletal locations and despite different embryonic origin (104). GWAS on SK-BMD have confirmed that genes involved in bone homeostasis of the skull are indeed largely the same as those regulating the appendicular skeleton, including genes identified using more distant phenotypes like from the heel GWAS (12, 15). This way, genes associated with craniosynostosis have also been associated with fracture risk and trabecular bone maintenance, as is the case for *EN1* (31). Despite the large similarity among the genes contributing to BMD of the skull and of BMD measured at other skeletal sites, SK-BMD studies illustrate distinctive regulatory mechanisms in the same genes and associated genetic networks (104, 106). Deciphering these regulatory mechanisms could help us understand (among other mechanisms) temporospatial shifts in gene expression among distinct skeletal sites and types of ossification.

Further, the prioritization of BMD genes associated with craniosynostosis for functional studies may highlight genes with osteoinductive and osteoanabolic properties. It will be essential to prove that identified craniosynostosis genes and family members are causal genes in BMD and understand why not all craniosynostosis genes are being identified through BMD-GWAS and whether they would belong to a different class of “bone-active” genes. Additionally, it will be interesting to validate the osteoinductive potential of craniosynostosis genes for the treatment of fracture healing and regeneration, especially for genes already known to act in the suture mesenchymal cell pool.

From a clinical perspective, the intersection between BMD-GWAS and craniosynostosis also alludes to the possibility of identifying new craniosynostosis genes, that could underlie the presentation of non-syndromic cases in which 80% of the genetic etiology remains unknown. To date, craniosynostosis GWASs are limited by sample size (typically in the hundreds). This is in contrast to the large numbers achieved by the SK-BMD GWAS, (with more than 50K samples and a proportional increase in the yield of identified loci). We advocate that clinicians in the field of craniosynostosis draw close attention to searching mutations in SK-BMD genes among patients whose genetic causes remain unsolved.

In conclusion, zebrafish now constitute the preferred model organism to validate genes overlapping craniosynostosis and osteoporosis, helping to elucidate the genetic overlap between the conditions. Such pleiotropy studies hold the potential to enhance our knowledge about the underlying biology of diverse skeletal conditions and potentially identify therapeutic targets. A glimpse into the near future depicts a considerable increase in the incorporation of zebrafish models for identifying causal genes and providing in-depth mechanistic evaluations with multiple translational opportunities to improve the care of patients with skeletal conditions. Similarly, researchers in the zebrafish field will incorporate more comprehensive genomic evaluations and innovative artificial intelligence approaches to speed phenotypic analyses. Last but not least, the implementation of cohesive platforms to access functional data from studies performed on zebrafish and standardization of methodologies to assess bone phenotypes in zebrafish will become a reality through multidisciplinary collaboration.

## Author contributions

EK: Conceptualization, drafting, writing, visualization, and editing. All authors have read and edited the manuscript. All authors contributed to the article and approved the submitted version.

## Funding

EK was supported by Versus Arthritis, grant #21937. SB: National Institute of Health-NIDCR grants R01 DE-16886 and R03 DE-031061.

## Acknowledgments

We acknowledge Dr. P. Eckhard Witten (University of Gent, Belgium) and his laboratory for providing images for **Figure 4**. We thank Dr John Kemp for proofreading the manuscript and for sharing critics. EK Thank Dr. Chrissy Hammond for her support. This article is based upon work from COST Action GEMSTONE CA18139 supported by COST (European Cooperation in Science and Technology) www.cost.eu.

## Conflict of interest

The authors declare that the research was conducted in the absence of any commercial or financial relationships that could be construed as a potential conflict of interest.

## Publisher’s note

All claims expressed in this article are solely those of the authors and do not necessarily represent those of their affiliated organizations, or those of the publisher, the editors and the reviewers. Any product that may be evaluated in this article, or claim that may be made by its manufacturer, is not guaranteed or endorsed by the publisher.

## Abbreviations

BMD, Bone mineral density; BMP, bone morphogenic protein; DXA, dual-energy X-ray absorptiometry; MAF, minor allele frequency; GEFOS, Genetic Factors for Osteoporosis; GOF, Gain-of-function; GWAS, Genome-Wide Association Studies; IMPC, International Mouse Phenotyping Consortium; IKMC, International Knockout Mouse Consortium; OBCD, Origins of Bone and Cartilage Diseases; SK-BMD, skull bone mineral density; WES, Whole-exome sequencing; WGS, Whole-genome sequencing; WHO, World Health Organization.
